# Opsonic and Protective Properties of Antibodies Raised to Conjugate Vaccines Targeting Six *Staphylococcus aureus* Antigens

**DOI:** 10.1371/journal.pone.0046648

**Published:** 2012-10-15

**Authors:** Clarissa Pozzi, Katarzyna Wilk, Jean C. Lee, Marina Gening, Nikolay Nifantiev, Gerald B. Pier

**Affiliations:** 1 Channing Laboratory, Department of Medicine, Brigham and Women's Hospital, Harvard Medical School, Boston, Massachusetts, United States of America; 2 N. D. Zelinsky Institute of Organic Chemistry, Russian Academy of Sciences, Moscow, Russia; University of Liverpool, United Kingdom

## Abstract

*Staphylococcus aureus* is a major cause of nosocomial and community-acquired infections for which a vaccine is greatly desired. Antigens found on the *S. aureus* outer surface include the capsular polysaccharides (CP) of serotype 5 (CP5) or 8 (CP8) and/or a second antigen, a β-(1→6)-polymer of *N*-acetyl-D-glucosamine (PNAG). Antibodies specific for either CP or PNAG antigens have excellent in vitro opsonic killing activity (OPKA), but when mixed together have potent interference in OPKA and murine protection. To ascertain if this interference could be abrogated by using a synthetic non-acetylated oligosaccharide fragment of PNAG, 9GlcNH_2_, in place of chemically partially deacetylated PNAG, three conjugate vaccines consisting of 9GlcNH_2_ conjugated to a non-toxic mutant of alpha-hemolysin (Hla H35L), CP5 conjugated to clumping factor B (ClfB), or CP8 conjugated to iron-surface determinant B (IsdB) were used separately to immunize rabbits. Opsonic antibodies mediating killing of multiple *S. aureus* strains were elicited for all three vaccines and showed carbohydrate antigen-specific reductions in the tissue bacterial burdens in animal models of *S. aureus* skin abscesses, pneumonia, and nasal colonization. Carrier-protein specific immunity was also shown to be effective in reducing bacterial levels in infected lungs and in nasal colonization. However, use of synthetic 9GlcNH_2_ to induce antibody to PNAG did not overcome the interference in OPKA engendered when these were combined with antibody to either CP5 or CP8. Whereas each individual vaccine showed efficacy, combining antisera to CP antigens and PNAG still abrogated individual OPKA activities, indicating difficulty in achieving a multi-valent vaccine targeting both the CP and PNAG antigens.

## Introduction

Effective vaccination against infections due to Staphylococcus aureus, one of the most common causes of both community-acquired and life-threatening nosocomial infections [1], [2] has a clear and high priority. Despite promising preclinical data obtained from protection studies in animals, vaccines that targeted S. aureus capsular polysaccharides (CP) type 5 (CP5) and type 8 (CP8) antigens [3], the iron-surface determinant B (IsdB) protein [4], [5], a monoclonal antibody to lipoteichoic acid, as well as an immune globulin selected from plasma donors with high titers of antibody to clumping factor A (ClfA) [6], all failed to protect patients against staphylococcal infections in phase III clinical trials [3], [7], [8].

One major issue in vaccine research for S. aureus infections is a lack of knowledge as to the target antigens and immune effectors that optimally protect humans against this pathogen. Thus, current attempts to develop vaccines are essentially empiric, utilizing examples from successful approaches for other pathogens, animal protection studies, and in vitro correlates such as opsonic killing or interference with binding of bacteria to target proteins. As a result of this approach, and given the multiple and redundant virulence factors of S. aureus, it might be logical to deduce that an effective vaccine may need to be composed of multiple bacterial components, potentially incorporating surface polysaccharides, toxoids, and cell-wall associated proteins.

Using empiric approaches derived from protective efficacy observed in animal studies of S. aureus infection, candidates for inclusion in a multi-component staphylococcal vaccine encompass the polysaccharide antigens poly-N-acetyl glucosamine (PNAG), expressed by >95% of strains [9], and CP5 and CP8, produced by ∼75% of strains. A key characteristic of the PNAG antigen, in terms of its vaccine potential, is that the immune response needed to elicit optimal opsonic and protective antibody is affected by the N-acetyl groups on the glucosamine constituents [10]. When native PNAG from S. aureus (>90% acetylated) was chemically treated to reduce acetylation to ∼15%, the de-acetylated PNAG glycoform (dPNAG) elicited opsonic and protective antibody against S. aureus [10] as well as other PNAG-producing pathogens [11], [12], [13]. In contrast, antibody specific to epitopes incorporating the acetylated glucosamine monomers on PNAG were poorly opsonic and not protective [10]. Notably, most humans (>95%) have high titers of natural antibody directed to the acetylated epitopes of native PNAG, and this antibody is poorly opsonic and not protective in animal models. Some, but not all, human infections with S. aureus induce opsonic antibodies to dPNAG [14], [15], and ∼3% of normal humans have natural dPNAG-specific opsonic antibody (unpublished finding). The validity of raising antibody to the deacetylated glycoform of PNAG to produce protective antibody was strongly validated in work that used a synthetic oligosaccharide composed of nine b-1-6-linked monomers of glucosamine (9GlcNH_2_) conjugated to tetanus toxoid (TT) as a vaccine. This glycoform engendered opsonic and protective antibody whereas the fully acetylated synthetic glycoform conjugated to TT, 9GlcNAc-TT, did not induce protective immunity. However, whether the antibodies elicited to the synthetic oligosaccharide would also interact in a negative manner with antibodies to CP5 or CP8 was not investigated.

Additional candidate components for a multi-valent vaccine for S. aureus include two cell wall-anchored proteins, IsdB and ClfB, both of which have shown protective efficacy in animals [4], [16]. Although a clinical trial of the IsdB antigen as a single vaccine component to prevent post-surgical wound infections following cardiothoracic surgery was terminated [7], IsdB might nonetheless contribute to a multi-component vaccine, as immunization with this antigen has protected mice against lethality and renal abscess formation [4], [17]. A ClfB vaccine was protective against nasal colonization [18]. Another component under clinical development is alpha-hemolysin (Hla), a secreted S. aureus toxin that causes pore formation in eukaryotic cells. Immunization with a genetically engineered non-toxic Hla (H35L) protein [19] protected against lethality and reduced infection severity from sublethal doses of S. aureus in models of systemic infection [20] and pneumonia [21], although trials of Hla-based vaccines in humans dating back to the 1930's have been of uncertain efficacy [22].

Overall, using the animal protection data to make judgments as to potential vaccine components that might be incorporated into a comprehensive, multi-component vaccine for *S. aureus*, wherein some additive or synergistic efficacy might be achieved, we have prepared 3 different conjugate vaccines using the polysaccharide antigens 9GlcNH_2_ (for PNAG), CP5 and CP8, and 3 proteins, Hla-H35L, IsdB and ClfB as carriers, wherein 9GlcNH_2_ was conjugated to Hla-H35L, CP5 was conjugated to ClfB and CP8 to IsdB, to evaluate immunogenicity in animals, in vitro activities including opsonic killing, toxin neutralization and inhibition of binding to human fibrinogen by ClfB along with protection in mouse models of S. aureus skin abscess formation, pneumonia, and nasal colonization.

## Materials and Methods

### Bacterial strains used

S. aureus isolates used were CP8 strain MN8 [23], CP5 strain Newman [24], and nontypable (NT) USA 300 MRSA strain LAC [25]. Isogenic variants lacking cap, ica, clfA, clfB, and isdB have been previously described [10], [26], [27], [28], [29], [30], [31], [32]. Mutants were constructed in spontaneous streptomycin-resistant (Sm^r^) isolates of wild-type S. aureus strains Newman [33] that were selected on tryptic soy agar (TSA) plates containing 0.5 mg/ml streptomycin as described before [16].

### Carbohydrate components of the vaccines

Purified CP5 and CP8 were prepared as described [32]. Synthetic 9GlcNH_2_-S-S was produced as described [34].

### Construction of recombinant rHla-H35L and rClfB expression vectors

To generate plasmid pHla, hla was PCR amplified from S. aureus strain DU1090 (pH35L) [19] with primers: 5′- CCCGGGCTCGAGAATGCCGCAGATTCTGATATTAATATTAAAACC-3′ and 5′CCCGGGGATCCTTTAATTTGTCATTTCTTCTTTTTCCCAATCGATTTTATATCT-3′.

To generate pClfB, *clfB* was amplified by PCR from S. aureus strain Newman with primers 5′-CCCGGGCTCGAGTCAGAACAATCGAACGATACAACGCAATCT-3′ and 5′-CCCGGGGGATCCAATCACCATCAGCACTTCCACCAC-3′. The primers for both targeted genes carried restriction sites for BamHI and XhoI at the 5′ and at the 3′ ends restriction sites for BamHI and XhoI, respectively. Amplified PCR fragments were cloned into the expression vector pET16b (Novagen) digested with BamHI and XhoI to yield pHla and pClfB and introduced into *E. coli* TOP10 cells and subsequently transformed into *E. coli* BL21(DE3).

Biologically active Hla was produced by *Escherichia coli* BL21 carrying the cloned gene in the vector pGEX 4TI [35], which was kindly provided by Pyong Park, Children's Hospital, Boston.

IsdB protein was produced by *Escherichia coli* TOP3 cells carrying plasmid pQE30iisdB [36] which was kindly provided by Prof. Tim Foster, Trinity College, Dublin.

### Purification of recombinant proteins

Expression of genes for active Hla, non-toxic Hla-H35L, ClfB and IsdB proteins was induced with 1 mM IPTG and the resultant His-tagged proteins purified by Ni2+ affinity chromatography as described previously [37] at the New England Research Center for Excellence Biomolecule Production Core Laboratory. Purities >95% by Coomassie-stained gels were achieved for all proteins (Figure S1).

### Preparation of conjugate vaccines

The synthetic oligosaccharide 9GlcNH_2_-S-S [34] (1.0 mg in 100 µl of 0.1 M sodium phosphate, 0.15 M NaCl, 10 mM EDTA, pH 8.0) was treated with washed Tris (2-carboxyethyl) phosphine hydrochloride **(**TCEP) disulfide reducing gel (200 µl of a 50% slurry in water). After incubation on a rotor rack at 22–24°C for 45 min, SH-derivatized oligosaccharides were separated from the gel by centrifugation, and the gel-immobilized TCEP was washed with the same pH 8.0 buffer (3×100 µl) and supernatants combined.

To activate the carrier protein, Hla-H35L (2 mg) was diluted in 200 µl of 0.1 M sodium phosphate, 0.15 M NaCl, 10 mM EDTA, pH 7.2 and a solution of N-hydroxysuccinimidyl-3- (bromoacetamido) propionate (SBAP, 1.3 mg in 40 µl of DMSO) added and incubated for 2 h at 22–24°C. Unreacted SBAP was removed using a PD-10 column in 0.1 M sodium phosphate, 0.15 M NaCl, 10 mM EDTA, pH 8.0 buffer, and the eluate concentrated to 400 µl. The SH-activated oligosaccharide was immediately combined with modified Hla-H35L protein (400 µl in pH 8.0 buffer), this mixture stirred 18–24 h at 22–24°C, then the conjugate separated from uncoupled components by gel filtration on Superose 6 prep-grade column. Fractions containing 9GlcNH2-Hla conjugate were pooled, concentrated and stored frozen at −20°C.

For the preparation of capsular polysaccharide conjugate vaccines, CP5 or CP8 (2.88 mg in 0.05 M of sodium borate pH = 9.22) were treated with 1-cyano-4-dimethylaminopyridinium solution (20 mg/ml) for 2 min at 20°C. The activated polysaccharides were immediately combined with the carrier proteins, ClfB or IsdB, respectively, (1.4 mg in sodium borate buffer, pH = 9.22), and the mixture stirred for 3 h at 20°C. The conjugated CP and carrier proteins were separated from uncoupled components by gel filtration on a Superose 6 prepgrade column followed by removal of unconjugated CP by passing the pooled fractions over a Ni-affinity column to retain the His-tagged ClfB or IsdB while allowing unconjugated CP antigens to flow through the column. Fractions containing CP5-ClfB and CP8-IsdB conjugates were pooled, concentrated and frozen at −80° C.

### Chemical analysis of the conjugate vaccines

9GlcNH_2_-Hla conjugate was analyzed for the oligosaccharide content using a hexosamine assay [38] with the 9GlcNH_2_ free oligosaccharide used as a standard. The CP5-ClfB and CP8-IsdB conjugate vaccines were analyzed by ELISA using the corresponding free, purified polysaccharide as standard. The proteins were measured with a Bradford assay using BSA [39] as standard.

### Antibody production using oligosaccharide conjugates

Rabbits were immunized subcutaneously with 10 µg (carbohydrate content) of the 9GlcNH_2_-Hla, CP5-ClfB and CP8-IsdB conjugates twice, one week apart, with an equivalent volume of incomplete Freund's adjuvant. On the third week, rabbits were immunized three times on alternate days with 5 µg (carbohydrate content) of the conjugate given intravenously in saline. Blood was taken two weeks after the final immunization and again two weeks apart three times.

### Antibody analysis by ELISA

Analysis of the antibody binding by ELISA to native PNAG was done as previously described [10]. A regression analysis formula was generated from linear values calculated by plotting the data as the log_10_ serum dilution versus the OD_405_ value [40]. The antilog of the dilution giving a value of zero was designated the serum antibody titer.

### Antibody analysis for opsonic killing activity

Antibody-dependent opsonic-killing (OPK) assays followed published protocols [10], [11], [14] with some changes. In place of neutrophils isolated from fresh human blood, we used an HL-60 cell line [41] that was kept at low passage (<3 months) and then exchanged for freshly thawed aliquots. In accordance with the protocol of Breitman et al, [42], maintenance of HL-60 cells was performed in L-glutamine-containing RPMI 1640 medium (Gibco Ltd., Carlsbad, Calif) supplemented with 15% heat-inactivated fetal bovine serum and a standard antibiotic-antimycotic mixture. The cells were kept in a 5% CO_2_ atmosphere at 37°C for four days and were then harvested. Under these conditions, HL-60 cultures can be maintained by diluting the cells with fresh medium to a density of 10^5^ viable cells/ml when the cell density reaches 10^6^ cells/ml. N,N-dimethylformamide (DMF) (0.8%) was used to induce granulocytic differentiation [41]. Optimal conditions for differentiation into neutrophils with 100 mM DMF occurred over a period of 5 days [43], [44], [45].

For the OPK assay, differentiated HL60 cells were washed in Hanks' Balanced Salt Solution (HBSS) lacking calcium chloride and magnesium chloride and resuspended in HBSS with these two divalent cations (HBSS^++^) supplemented with 0.1% gelatin (Sigma). Trypan blue staining was used to differentiate dead from live leukocytes, and the final cell concentration adjusted to 2.5×10^7^ HL60 cells per ml. The complement source was rabbit serum (Invitrogen) diluted 1∶10 in HBSS^++^ +1% Gelatin, which was adsorbed at 4°C for 30 min with continual mixing with the target S. aureus bacteria resuspended from a pellet containing 10^9^ CFU. After adsorption, the complement solution was centrifuged and filter sterilized.

To achieve antigenic specificity in the OPK assay, polyclonal sera were adsorbed with ∼10^10^ CFU/ml of either S. aureus MN8Δ*ica* [10] to remove all antibodies to staphylococcal surface antigens except those to PNAG, or S. aureus Newman Δ*cap5*, when testing OPKA against a CP5 strain, or S. aureus MN8 Δ*cap8*, when testing OPKA against a CP8 strain. These latter two adsorptions removed antibodies to all antigens except the corresponding CP [30], [31]. Additionally, a double gene deletion in S. aureus MN8 lacking both the ica and cap8 loci (strain MN8 Δ*ica*+Δ*cap*8) was used to adsorb antibodies to all antigens except those to PNAG and CP8 that might be present in an immune serum. USA 300 strain LAC, which is CP-negative, was used as a target strain to detect the opsonic killing activity specific to ClfB and IsdB cell-anchored surface proteins. To produce ClfB and IsdB antibodies, CP5–ClfB polyclonal serum was adsorbed with Newman Δ*clfB* strain and CP8-IsdB antiserum was adsorbed with Newman Δ*isdB* supplemented with 2 µg of CP8 to mask the CP8 mediated OPKA. The test sera were adsorbed at 4°C for 30 min, the bacterial cells removed by centrifugation, and the test sera filter sterilized.

The bacterial strains to be evaluated for phagocyte-dependent killing activity of antibody in immune sera were grown under different conditions associated with expression of surface antigens. For CP and PNAG antigen expression, S. aureus strains were grown overnight in Columbia broth supplemented with 2% NaCl (CSB). For testing of OPKA against the ClfB antigen, bacteria were grown to early log phase in CSB, when antigen expression is maximal. For testing the OPKA against IsdB, bacterial cells were grown in low-iron medium needed to induce antigen expression, which consisted of RPMI supplemented with 1% casamino acids. In all cases, the culture was adjusted to an OD_650_ of 0.4 using HBSS^+/+^ +1% gelatin (∼6×10^8^ CFU/ml), and then a further 1∶300 dilution in HBSS^+/+^ +1% gelatin made for use in the killing assay to achieve a final bacterial concentration of ∼2×10^6^ cfu/ml.

The OPK assay was performed by mixing 100 µl (each) of the HL60 suspension, complement source, dilutions of test sera (made in HBSS^+/+^ +1% gelatin) and target bacteria (HL60 cell to bacterial cfu ratio ∼12.5∶1). The reaction mixture was incubated on a rotor rack at 37°C for 90 min; samples were taken at time zero and after 90 min. A 10-fold dilution was made in TSB with 0.5% Tween to inhibit bacterial aggregation, and samples were plated onto TSA plates. Tubes lacking any serum and tubes with normal rabbit serum (NRS) were used as controls, as were tubes containing serum and complement but lacking HL60 to control for potential aggregation of bacteria by the antibody, which would reduce the apparent CFU counts at the end of the assay. At the concentrations of antisera used in the opsonic killing assay, there was no reduction in CFU of >10% in samples lacking phagocytes but containing antibody and complement, indicating little agglutinating activity of the antibody to the target antigens.

The percentage of killing was calculated by determining the ratio of the number of CFU surviving in the tubes with bacteria, leukocytes, complement, and sera to the number of CFU surviving in tubes containing normal rabbit sera, bacteria, complement, and leukocytes. Killing rates of >30% were considered biologically significant as this level represents the upper limit of the percentage reduction in CFU that occurs in the opsonic killing assay with normal sera lacking antibody to *S. aureus*, due to normal variation in CFU counts in this assay.

### Rabbit red blood cell (RRBC) hemolysis assay

Purified, active Hla at a final concentration of 6.25 nM was incubated with 2 fold dilutions of either normal or immune rabbit polyclonal serum raised against 9GlcNH_2_-Hla and a suspension of 12.5% rabbit red blood cells (RRBC) in PBS. After incubation for 1 h at 20°C, the samples were centrifuged at 2,000 rpm for 10 min. Supernatants were removed and the absorbance due to released hemoglobin was measured at 475 nm. The percentage of hemolysis was calculated using as a denominator the OD_475_ nm reading from an equivalent number of RBC that had been lysed in 1% Triton X-100 to indicate 100% lysis. The 50% inhibition titer was calculated using non-linear regression for sigmoidal curves with variable slopes (PRISM 4 software).

### Bacterial adherence to immobilized fibrinogen

Binding of S. aureus cells to immobilized fibrinogen was performed as described previously [46]. Human fibrinogen was diluted in PBS to 10 µg/ml, and 100 µl was used to coat 96-well flat-bottomed ELISA plates incubated overnight at 4°C. The plates were washed in PBS and blocked for 2 h at 37°C with 5% BSA in PBS. A 20 ml culture of S. aureus Newman ΔclfA was grown to an OD 600 of 0.5, centrifuged, washed in PBS and resuspended to an OD_600_ nm of 1.0 in PBS. The cell suspension was mixed with two fold dilutions of NRS or antibody to the CP5-ClfB conjugate vaccine that were absorbed twice with S. aureus Newman ΔclfB to leave only antibodies to the ClfB antigen in the control and test sera. The bacteria and serum mixtures were added to the fibrinogen-coated plates and incubated 2 h at 37°C. The cells were washed in PBS and fixed by adding 100 µl of 25% aqueous formaldehyde, and incubating at room temperature for 15 min. The plates were then washed gently, stained with crystal violet, and washed again. The stained cells were resuspended in 5% acetic acid, and the OD_570_ nm read on an ELISA reader. The 50% inhibition of binding titer was calculated using non-linear regression for sigmoidal curves with variable slopes (PRISM 4 software).

### Murine pneumonia and skin infection models

Animal experiments were approved by the Harvard Medical Area Institutional Animal Care and Use Committee. For mouse lung infections, S. aureus strain Newman was grown at 37°C on Columbia salt agar for 18 h then inoculated into PBS to an OD_600_ nm of 0.4. Culture aliquots were centrifuged and washed in PBS, and the bacterial cells suspended in PBS to achieve 2×10^8^ CFU per 20 µl for Newman and 4×10^8^ CFU for PS80 and USA 300 LAC. FVB mice (Jackson Laboratory) 4–5 weeks old were anesthetized and immunized with 10 µl of NRS or antisera to 9GlcNH_2_-Hla per nostril twenty-four and four h before challenge with S. aureus. Twenty-four h after infection mice were sacrificed and lungs were excised, weighed and homogenized for enumeration of the lung bacterial load.

For evaluation of survival in a moribund/lethal infection model, S. aureus strains LAC (USA 300) and LAC Δ*ica* were grown at 37°C on Columbia salt agar for 18 h then inoculated into TSB supplemented with 1% glucose and grown to an OD_600_ nm of 1.0 with shaking at 250 rpm. Cultures were centrifuged and washed in PBS, and the bacterial cells suspended in PBS to achieve 5×10^8^ CFU per 20 µl. Four weeks old FVB mice were anesthetized by injection of ketamine and xylazine and immunized with 10 µl of NRS or antisera to 9GlcNH_2_-Hla per nostril twenty-four and four h before challenge with S. aureus. Survival was monitored over time and moribund animals sacrificed and counted as dead for purposes of data analysis using the Log-rank test.

For mouse skin infections, S. aureus strains Newman, PS80 or LAC were grown at 37°C on Columbia salt agar plates for 18 h then suspended into PBS to an OD_600_ nm of 0.4. Culture aliquots (10 ml for Newman and 1 ml of PS80 and LAC) were centrifuged and re-suspended in 10 ml of a 1∶1 ratio of PBS and Cytodex micro-carrier beads, 67–80 µm (Sigma) as described [47]. To test the protection of antibodies to ClfB against S. aureus LAC in the skin infection model, bacteria were grown at 37°C in CSB to an optical density at 600 nm of 0.5. 10 ml of culture was centrifuged and re-suspended in 10 ml of 1∶1 ratio of PBS and micro-carrier beads.

Abscess formation was induced in mice by sc infection with S. aureus. FVB mice (3–5 weeks old) were injected by the intraperitoneal (IP) route with 0.3 ml of either NRS or immune sera to 9GlcNH2-Hla, CP5-ClfB or CP8-IsdB 24 and 4 h prior to S. aureus infection. One-hundred µl of the challenge bacteria was injected into both flanks of a mouse and after 72 h the animals euthanized and the abscesses removed, dissected, homogenized and diluted in TSB for bacterial enumeration. For the analysis of interaction between antibodies to PNAG and CP antigens, a mix of antisera (100 µl of each) to 9GlcNH2-Hla, CP5-ClfB or CP8-IsdB was injected IP 24 and 4 h and before challenge with S. aureus. Controls received 300 µl of NRS.

### Nasal colonization model

FVB mice 3–5 wks old were passively immunized IP with 0.3 ml of rabbit polyclonal antibodies raised to CP5-ClfB or with NRS as the control 14 h prior to inoculation with 10^9^ CFU of S. aureus Newman resistant to streptomycin (Sm^r^) as described [16]. The culture was grown at 37°C for 28 h on TSA and then subcultured into 250 ml of TSB until an OD_600_ nm of 0.4 was achieved. The culture was washed in PBS and suspended in 1 ml of PBS (1×10^9^ CFU per 20 µl). Animals were challenged by the IN route with 20 µl of S. aureus Sm^r^ Newman as described previously [16], [33]. The mice were euthanized 6 days after bacterial challenge. The noses were excised and homogenized in 1 ml of TSB. The suspension was plated on 0.5 mg streptomycin/ml to determine the number of S. aureus Newman Sm^r^ present per nose.

### Statistical analysis

Statistical analyses were performed using the Prism software. The Mann-Whitney non-parametric two-tailed U test for two sample comparisons was used to analyze paired data. Non-parametric ANOVA was used for multi-group comparisons and the Dunn Procedure for post-hoc pair-wise comparisons.

## Results

### Conjugate vaccine production

Recombinant, His-tagged non-toxic Hla (H35L), ClfB and IsdB proteins were synthesized from DNA in the pET16b expression vector (Novagen) using the T7 polymerase for transcription in *E. coli* BL21 (DE3) cells (Figure S1). The His tag was left on the proteins. These three recombinant proteins were then conjugated, respectively, to 9GlcNH_2_, CP5, or CP8, as described in Materials and Methods. Molecular sieve chromatography was performed to separate the 9GlcNH_2_ oligosaccharide conjugated to the carrier protein from the unconjugated oligosaccharide. However, it was not possible to ascertain how much, if any, unconjugated protein was present in the conjugate vaccine because the difference in size between 9GlcNH_2_-Hla conjugated and unconjugated Hla carrier protein was too small to reliably separate by size-exclusion chromatography. For the CP-conjugated antigens, molecular sieve chromatography was useful for separating conjugated and unconjugated protein, and Ni-affinity columns were useful for separating protein-conjugated CP from unconjugated CP.

### Rabbit immune responses to 9GlcNH2-Hla, CP5-ClfB and CP8-IsdB conjugate vaccines

Rabbits were immunized with each conjugate vaccine separately so that the production of antibody to the six antigens could be readily evaluated, individual as well as combined opsonic and/or protective activity to specific antigens could be tested, the potential for interference in antibody production or activity by having antigens to both PNAG and CP in the same vaccine was avoided, and sufficient amounts of consistent reagents for testing immunity in multiple animals and different sites of infection would be available. This approach does however, exclude any cellular immune component that could contribute to protective immunity following active vaccination.

**Figure 1:**
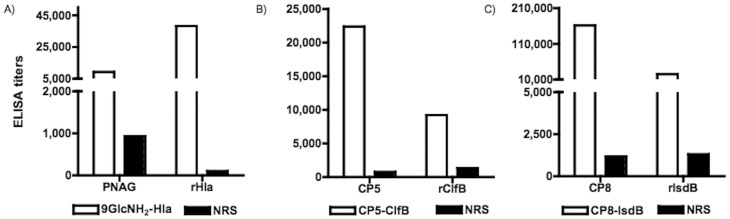
Immune response of rabbits immunized with 9GlcNH_2_-Hla, CP5-ClfB and CP8 IsdB conjugate vaccines. (a) ELISA titers to PNAG and recombinant Hla protein. (b) ELISA titers to CP5 and recombinant ClfB protein. (c) ELISA titers to CP8 and recombinant IsdB protein. Sera were obtained from rabbits immunized with two subcutaneous doses of the conjugate vaccines equivalent to 10 µg of carbohydrate and three I.V. doses corresponding to 7.5 µg of carbohydrate.

Immunization with the 9GlcNH_2_-Hla conjugate elicited high titers of antibody to native PNAG isolated from S. aureus and to the rHla carrier protein (Fig. 1a). Thus, although these antibodies were generated against epitopes lacking any acetates on the glucosamine oligosaccharide constituents, they nonetheless bound to the highly acetylated native form of the polysaccharide that is the dominant glycoform on bacterial surfaces [34]. Antisera against CP5-ClfB and CP8-IsdB had high antibody titers to native polysaccharides CP5 and CP8 and to the carrier proteins ClfB and IsdB (Figs. 1B and 1C).

In order to determine if, following conjugation with the polysaccharides, the carrier proteins (Hla, ClfB and IsdB) retained conformations associated with induction of antibodies recognizing the native proteins, appropriate anti-toxic, anti-adhesive or binding assays were performed with each individual antiserum.

**Figure 2:**
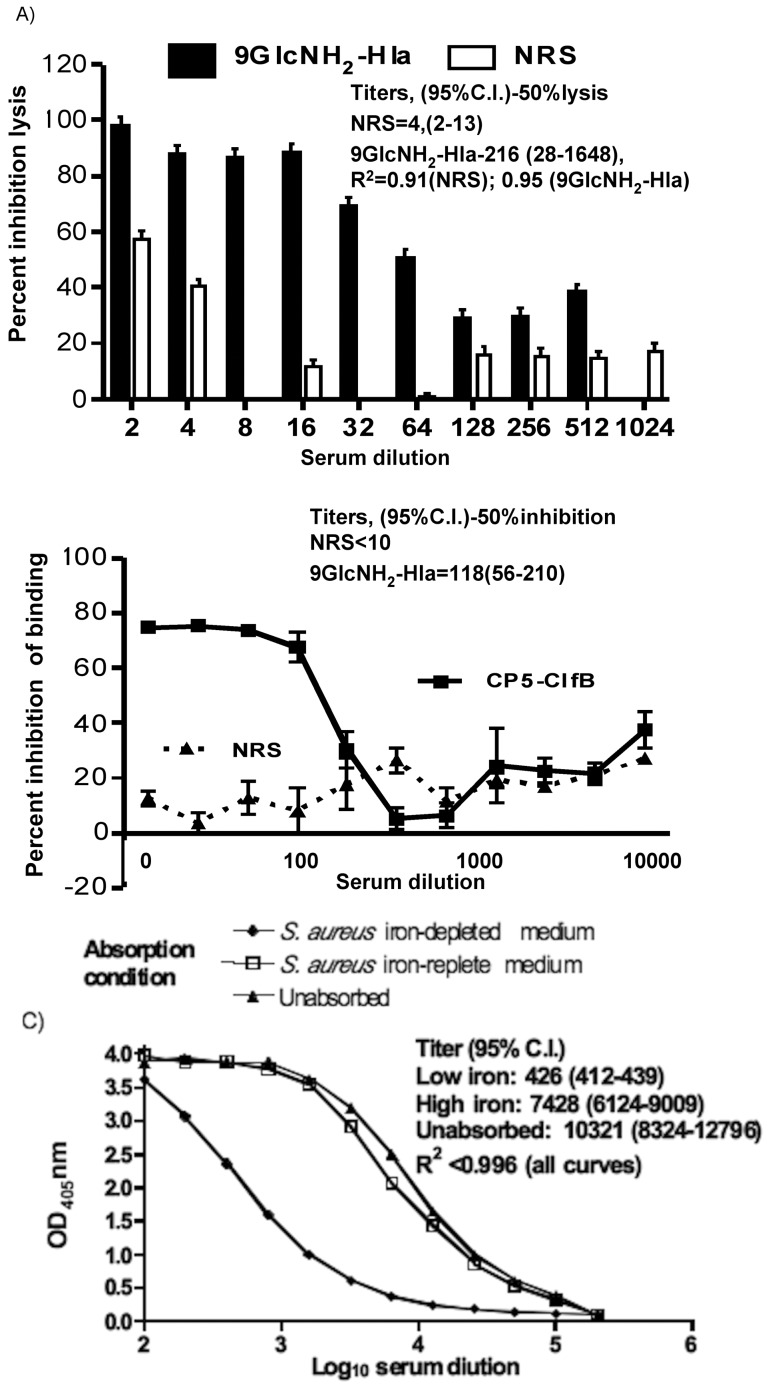
Effect of antisera to the carrier proteins Hla, ClfB and IsdB on their biological activities. (a) Rabbit antisera raised to 9GlcNH_2_-Hla inhibited lysis of rabbit red blood cells (RRBC) due to *S. aureus* hemolysin A. Percent hemolysis was calculated using as the denominator the haemoglobin absorbance at 475 nm obtained following lysis of control RBC in 1% Triton X-100. Lack of overlap in the 95% C.I. between the 50% lysis titers indicates difference significant at P<0.05. Bars represent mean of triplicates. (b) Inhibition of binding of *S. aureus* Newman Δ*clfA* due to antibody raised to CP5-ClfB to immobilized fibrinogen. Percent inhibition of binding was calculated using as the denominator the OD_650_ nm values obtained in the control with no serum. Lack of overlap in the 95% C.I. between the 50% lysis inhibition titers indicates difference significant at P<0.05. Lines represent mean of triplicates. (c) ELISA titers to purified IsdB protein in intact antisera or antisera absorbed with *S. aureus* cells grown in an iron depleted medium to induce IsdB, or in an iron-replete medium to repress IsdB synthesis. Lines represent mean of duplicates.

We evaluated the antibodies to 9GlcNH_2_-Hla for neutralization of the toxic activity of native Hla in a RRBC hemolysis assay. Purified native Hla (6.25 nM) was incubated with two fold dilutions of either NRS or antisera raised to 9GlcNH_2_-Hla and 12.5% RRBC. The dilution of serum (i.e., titer) needed to reduce hemolysis by 50% was calculated by non-linear regression and was found to be 4 (95% C.I. 2–13) for NRS and 216 (95% C.I. 28–1648) for the antiserum raised to 9GlcNH_2_-Hla (P<0.05, Fig. 2a).

It has been previously reported that in the absence of ClfA, which is the major *S. aureus* fibrinogen binding protein, S. aureus can bind immobilized fibrinogen through a ClfB-dependent mechanism [48]. To determine the functional activity of antibodies raised to the CP5-ClfB conjugate, we tested the inhibition of *S. aureus* adherence to fibrinogen using S. aureus strain Newman lacking the *clfA* gene (Δ*clfA*), grown to an OD_600_ nm of 0.5. *S. aureus* D*clfA* was incubated with NRS or antisera raised to CP5-ClfB, then added to fibrinogen-coated plates. The NRS was unable to inhibit binding of *S. aureus* Newman D*clfA* by >20% (titer <10) while antibody to CP5-ClfB had a 50% inhibitory titer of 118 (95% C.I. 56–210; P<0.05, Fig. 2b).

Iron surface determinant B (IsdB) plays a role in the acquisition of heme iron through the binding to hemoglobin, and it is only expressed under limited iron conditions, such as those that occur during infection in mammalian hosts. The binding specificity of antisera to CP8-IsdB antibodies was measured in an ELISA using purified IsdB protein and either intact antisera or antisera absorbed with S. aureus cells grown in low iron to induce IsdB or high iron to repress IsdB. The intact antiserum and serum absorbed with S. aureus MN8 grown in high iron both bound comparably to purified IsdB (Fig. 2c), indicating no loss of antibody from this absorption. However, antibody binding to IsdB was markedly reduced when the antiserum was absorbed with S. aureus MN8 grown in IsdB-inducing iron deficient conditions. The calculated end-point titer of the serum after absorption with S. aureus grown in low iron was significantly lower than that of the unabsorbed antibodies or antibodies absorbed with S. aureus grown in high iron (P<0.001, Fig. 2c), indicting antibodies raised to CP8-IsdB recognized the native IsdB protein on the bacterial surface.

### Opsonic killing activity of the antisera

We next used polyclonal rabbit antibody raised to 9GlcNH2-Hla, CP5-ClfB and CP8-IsdB to measure OPKA against S. aureus strains Newman (CP5), PS80 (CP8) and USA 300 LAC (CP-) in the presence of granulocytes (differentiated-HL60 human pro-myelocytic cells) and rabbit complement (C′).

**Figure 3:**
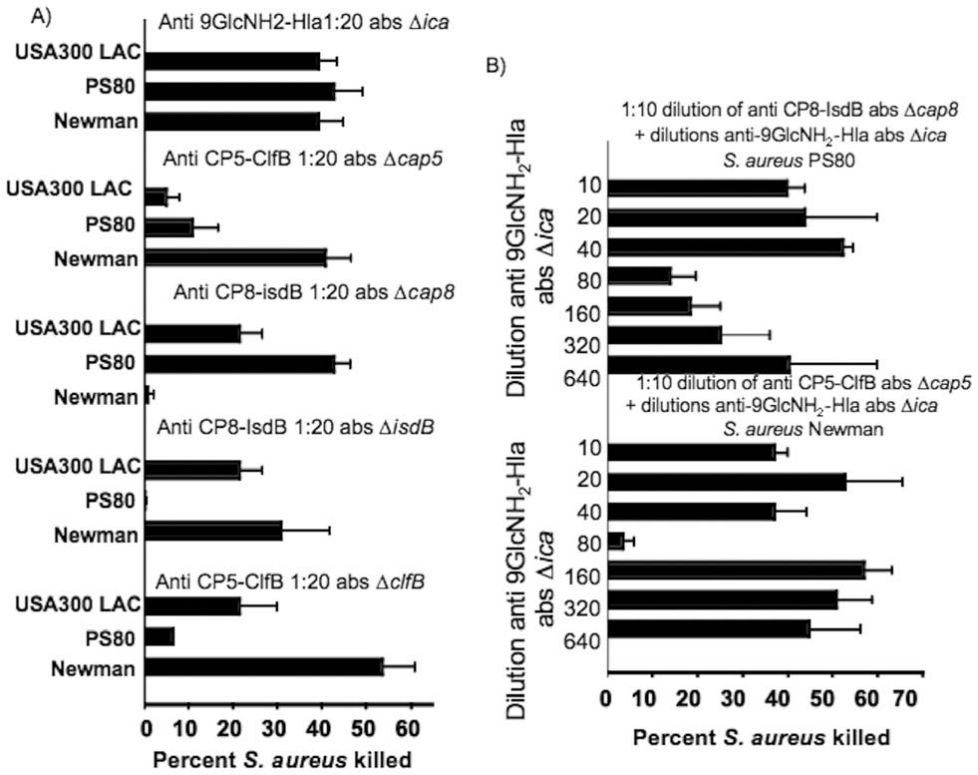
Opsonic killing activity in rabbit sera raised to 9GlcNH_2_-Hla, CP5-ClfB and CP8-IsdB conjugate vaccines. (A) Killing at a serum dilution of 1∶20 of *S. aureus* strains USA 300 LAC, PS80 and Newman with antisera raised to 9GlcNH_2_-Hla, CP5-ClfB and CP8-IsdB. Sera were absorbed for antigenic specificity as follows: antisera to CP8-IsdB were absorbed with strain Newman Δ*isdB* grown to stationary phase in RPMI medium with 1% casamino acids to make the serum specific to IsdB; antisera to CP5-ClfB were absorbed with strain Newman Δ*clfB* grown to log phase in CSB to make the serum specific to ClfB. (B). Opsonic killing of *S. aureus* strains PS80 and Newman when one antiserum was held constant (CP5-ClfB or CP8-IsdB) and the second one (9GlcNH_2_-Hla) diluted as indicated. Sera were made antigen-specific by absorption as indicated on figure. Bars represent means of triplicate samples, and the percent *S. aureus* killed was compared to normal rabbit serum. Controls lacking complement or PMN had <10% OPKA (not shown).

Animals immunized with the 9GlcNH_2_-Hla conjugate had opsonically-active antibody that mediated killing of all three PNAG-producing S. aureus strains Newman, PS80 and USA 300 LAC (Fig. 3a). Prior results [14], [15], [34], as well as laboratory experience, has found that sera diluted 1∶10 with PNAG-specific OPKA killing of <30% do not mediate protection in experimental animal infections. The rabbit antisera deemed to have a positive OPKA all showed antigen-specific bacterial killing >35% in a 1∶20 serum dilution that was reduced to <30% when sera were diluted from 1∶80 to 1∶640, but for clarity we are only showing the activity in the 1∶20 serum dilutions (Fig. 3a). In the OPKA using cells grown to stationary phase in CSB, which promotes CP expression, the antibodies raised to CP5-ClfB showed killing activity against S. aureus CP5 strain Newman but not against CP8 strain PS80 or CP^−^ USA 300 LAC (Fig. 3a). Similarly, antibodies raised to CP8-IsdB showed killing activity against S. aureus CP8 strain PS80 but not against CP5 strain Newman although a low opsonic activity was found against CP- USA 300 LAC (21%). As this could be due to antibody to IsdB, we next tested the OPKA of the antiserum to CP8-IsdB following absorption of this serum with S. aureus Newman D*isdB* to remove antibody to CP5 but retain antibody to IsdB. For this assay we also grew the target bacterial cells in a low-iron medium to enhance IsdB production. Here we found almost as much OPKA in this absorbed serum against CP^−^ strain LAC as there was in the unabsorbed serum raised to CP8-IsdB. Similarly, a low level of killing of S. aureus Newman was achieved with this antiserum, but not against strain PS80, indicating that antibody to IsdB can mediate a modest amount of OPKA against some S. aureus strains.

We also found the antiserum raised to CP5-ClfB, following absorption with S. aureus Newman D*clfB* to remove antibody to CP5, still had high killing activity (53%) against S. aureus strain CP5 Newman grown to early log phase (Fig. 3a) but not against PS80 or LAC (≤20% killing, not considered significant). In early log phase CP antigen production is low to absent [49], indicating that for strain Newman, depending on the phase of growth, antibody to either CP5 or ClfB can mediate OPK. Presumably the low level of killing of strains LAC and PS80 by antibody to ClfB reflects either poor expression of this antigen by these strains or perhaps other factors made by these strains that interfere with OPK mediate by antibody to ClfB. As with antibody to IsdB, OPK of different S. aureus strains is variable when using antibody to ClfB.

### Interference of opsonic killing mediated by mixtures of antibody to CP and PNAG antigens

Protective antibodies to both PNAG and *S. aureus* CP antigens must have in vitro OPKA to mediate in vivo protection in experimental models [10], [14], [15], [49]. Our group has previously shown that OPK of antibody induced in different rabbits by conjugate vaccines specific to either CP or PNAG antigens is high, but when mixed together they interfere with each other's OPKA due to a charge-based idiotype-anti-idiotype binding of the Fab regions of these antibodies [15]. This also abrogates the protective activity in skin infection and bacteremia models when the individually protective antibodies are mixed together [15]. However, these prior results were generated using a chemically deacetylated glycoform of native PNAG wherein about 15% of the glucosamine residues remained acetylated. Moreover, tetanus toxoid was used as the carrier protein for all three of the carbohydrate antigens. To ascertain if using the fully non-acetylated synthetic glycoform of PNAG, 9GlcNH_2_ along with conjugating each carbohydrate to distinct carrier proteins as vaccines could alter the induction of interfering antibodies, we next mixed together the polyclonal rabbit antisera raised to CP5-ClfB or CP8-IsdB with antibody to 9GlcNH_2_-Hla to determine if interference in OPKA against S. aureus strains Newman and PS80 still occurred. A 1∶20 dilution of rabbit antisera raised to 9GlcNH_2_-Hla showed antigen-specific OPKA >50% (Fig. 3b) that was lost when this serum was mixed with 1∶10 dilutions of antisera to either CP5-ClfB or CP8-IsdB (Fig. 3b). Interference disappeared as decreasing amounts of the antiserum to the CP surface polysaccharides were added. The interference when antibody to 9GlcNH_2_-Hla was mixed with antibody to CP8-IsdB was stronger than when mixed with antibody to CP5-ClfB, a phenomenon we noted previously wherein antibody to CP8 gives rise to stronger interference in OPK than does antibody to CP5 [15]. Thus the use of the synthetic oligosaccharide to PNAG and distinct carrier proteins did not overcome the interference in OPKA found when antibodies to this antigen were mixed with antibodies to *S. aureus* CP antigens.

### Passive immunization with antibody to 9GlcNH_2_-Hla protects mice from *S. aureus* pneumonia

**Figure 4:**
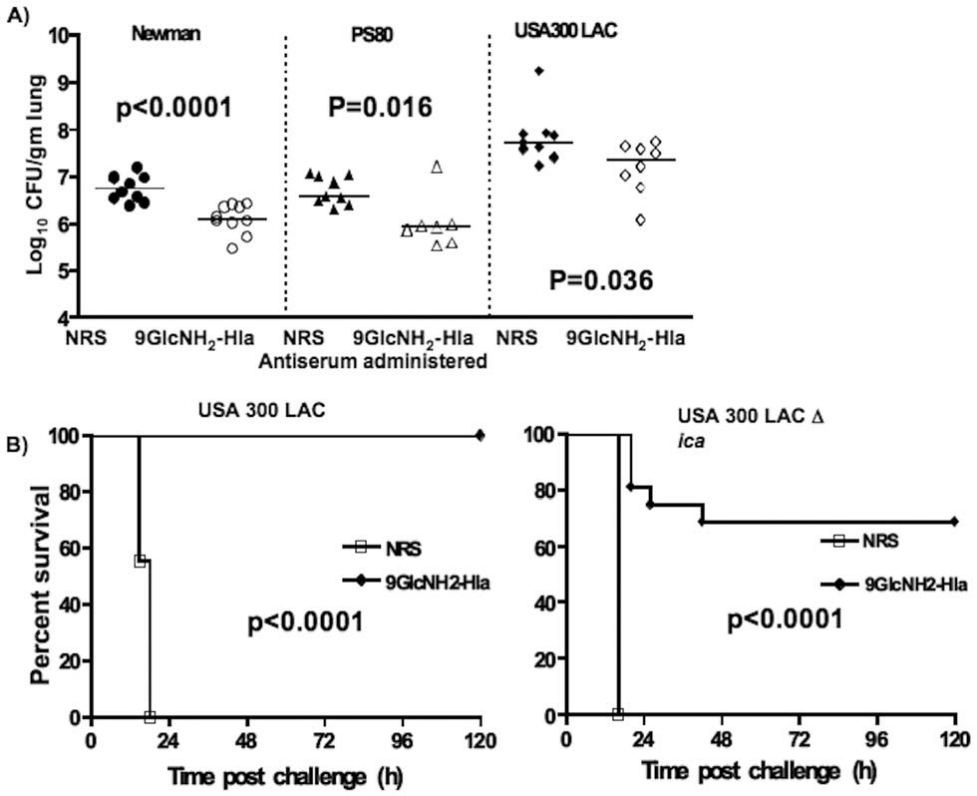
Passive immunization with 9GlcNH_2_-Hla antibodies in a murine model of *S. aureus* pneumonia. (a) Mice were vaccinated i.n with 20 µl of 9GlcNH_2_-Hla antisera or NRS 24 h and 4 h prior to infection with indicated *S. aureus* strains (4×10^8^ CFU/mouse). Bacterial burden was recorded 24 h post-infection. Points indicate cfu/lung of individual mice, lines the group median; p values: Mann-Whitney test. (b) Kaplan–Meier survival curve of 4 week old FVB mice vaccinated i.n with 20 µl of 9GlcNH_2_-Hla antisera or NRS 24 h and 4 h prior to infection with indicated *S. aureus* strains (6×10^8^ CFU/mouse). Survival was monitored at least twice a day over a 5 day period and data analyzed using the Log-rank test to derive the p values.

Several reports over the years [20], [21] described that transfer of Hla-specific antibodies protects naïve animals against S. aureus lethality and lung infection. To examine the ability of antibody to 9GlcNH_2_-Hla to prevent pneumonia induced by S. aureus grown to either the logarithmic or stationary phases, we passively immunized 3–5 week old FVB mice with 20 µl of either NRS or rabbit antibody to 9GlcNH_2_-Hla via the intranasal route (i.n.) route 24 h and 4 h before i.n. challenge with 2×10^8^ CFU of S. aureus Newman. Animals were sacrificed 24 h later and lungs removed and bacterial loads determined. Compared to the NRS control, passive immunization with 9GlcNH_2_-Hla antibodies caused a significant reduction in the staphylococcal load for animals challenged with *S. aureus* strain Newman strain grown to either the logarithmic (P = 0.015) (not shown) or stationary phases (P<0.0001) (Fig. 4 A), with a greater effect seen when the organisms grown to stationary phase were used for challenge.

Animals challenged with 4×10^8^ CFU of S. aureus PS80 or USA 300 strain LAC in stationary phase also had a reduction of CFU recovered from the lungs in the immune serum immunized groups compared to the NRS groups (P = 0.016 and P = 0.036, respectively).

To determine the contribution of the antibody to the PNAG antigen or the Hla hemolysin in reducing bacterial counts during *S. aureus* lung infection, we challenged 10 FVB mice passively given 20 µl of either NRS or antibody to 9GlcNH_2_-Hla with 4×10^8^ CFU of S. aureus USA 300 strain LAC unable to make PNAG (Δ*ica*) grown to stationary phase. Animals were sacrificed 24 h later and lungs removed and bacterial loads determined. Compared to the NRS control, passive immunization with 9GlcNH_2_-Hla antibodies did not show a significant reduction in the CFU recovered from the lungs (data not shown), suggesting that the Hla-antibodies are not involved in reducing *S. aureus* bacterial counts in the lungs.

As antibodies to Hla might augment overall survival from *S. aureus* lung infection without reducing bacterial levels, we passively transferred antibody to 9GlcNH_2_-Hla or NRS to mice 24 h and 4 h before i.n. challenge with a higher, lethal dose of 6×10^8^ CFU of S. aureus USA300 LAC or USA 300 LAC Δ*ica* strains grown to logarithmic phase. Survival (moribund or lethal) over the next 120 h was monitored (Fig. 4B). Animals challenged with USA 300 strain LAC had 100% survival if given antibody to 9GlcNH_2_-Hla (Fig. 4B) whereas 100% of mice given NRS died. A lower level of protection (70%) was achieved in mice infected with *S. aureus* strain USA 300 LAC Δ*ica* that had been given antibody to 9GlcNH_2_-Hla where all of the animals given NRS died within 48 h following challenge (P<0.0001). These data suggested that the combination of antibody to both PNAG and Hla had a somewhat better ability to protect mice against pneumonic death than did antibody to Hla alone, which would have been the effective activity in mice challenged with PNAG-negative *S. aureus* LAC D*ica*.

### Protective efficacy against *S. aureus* in a murine skin abscess model

**Figure 5:**
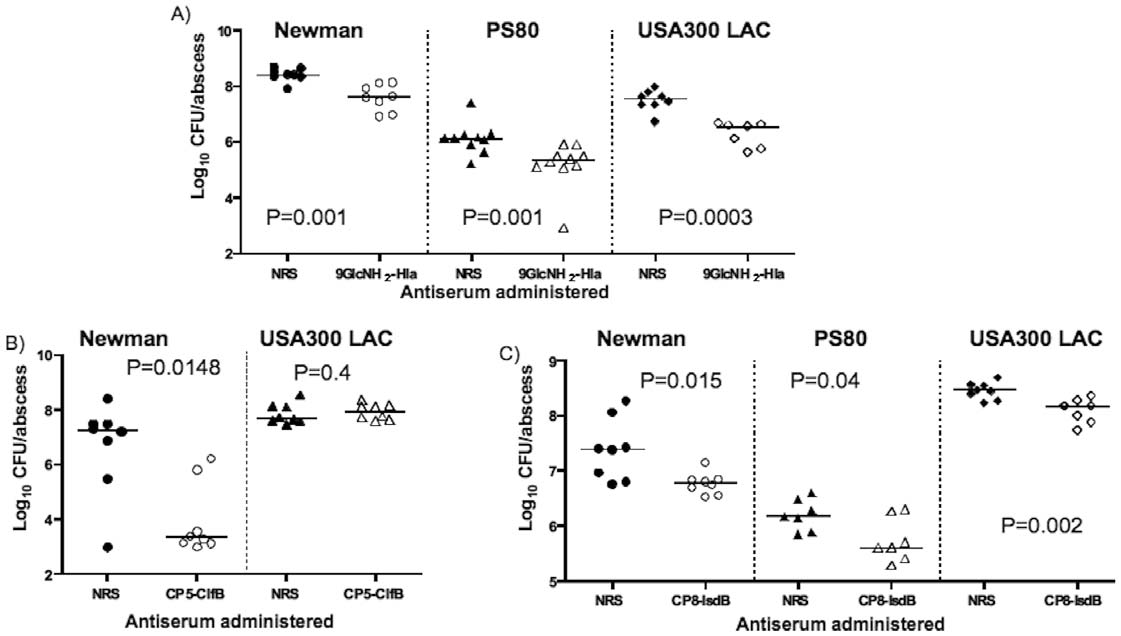
Protective efficacy of 9GlcNH_2_-Hla, CP5-ClfB or CP8-IsdB antibodies against skin abscess infection by *S. aureus*. Antisera (300 µl) were administered IP 24 h and 4 h prior to injection of skin with indicated *S. aureus* strain mixed with 60–87 µm dextran beads. Points indicate cfu/individual abscess, lines the group median, (A) Results following immunization with 9GlcNH_2_-Hla antisera or NRS and challenge with *S. aureus* Newman, PS80 or USA 300 LAC. (B) Results following immunization with CP5-ClfB antisera or NRS and challenge with *S. aureus* Newman, or USA300 LAC. (C) Results following immunization with CP8-IsdB antisera or NRS and challenge with *S. aureus* Newman, PS80 or USA300 LAC; (p values determined by Mann-Whitney test.

In a murine model of skin abscesses elicited by inoculating the bacteria subcutaneously along with small (60–87 µm) dextran beads [47], IP injection of 300 µl of immune sera against 9GlcNH_2_-Hla 24 h and 4 h prior to infection resulted in significant reductions in the bacterial CFU/abscess produced by S. aureus strain Newman (P = 0.0011), PS80 (P = 0.0007), or LAC (P = 0.0003) when compared with animals given NRS (Fig. 5a).

Antibodies to CP5-ClfB reduced the mean CFU/abscess recovered from mice challenged with CP5 strain Newman (P = 0.015) compared to the mice given NRS but did not reduce bacterial burdens when the animals were infected with a CP^−^ strain LAC (P = 0.38) (Fig. 5b), suggesting antibody to ClfB was not providing much protection is this setting.

The CP8-IsdB antiserum significantly reduced the CFU/abscess formed by CP8 strain PS80 (P = 0.04) (Fig. 5c), as well as those formed by S. aureus strains Newman (CP5) or LAC (CP^−^) (P = 0.0148 and P = 0.0019, respectively) (Fig. 5c). These findings suggest that not only are the antibodies to CP8 protective against S. aureus skin infections caused by CP8 strains, as previously shown [15], but also antibodies to IsdB have protective efficacy in this murine skin abscess model.

**Figure 6:**
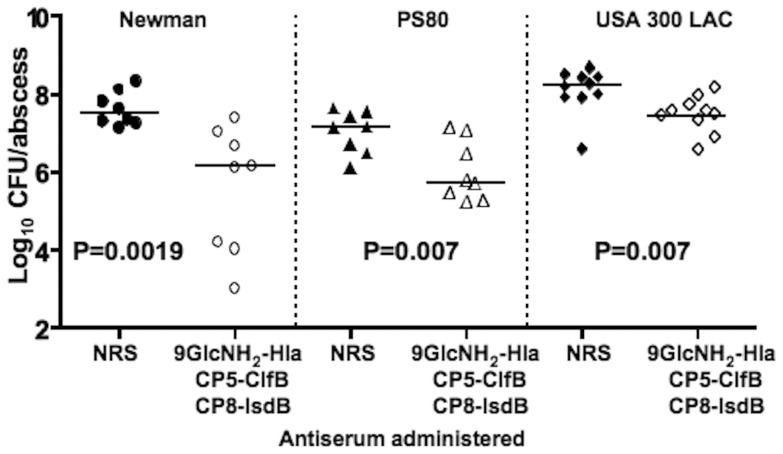
Protective efficacy of 9GlcNH_2_-Hla, CP5-ClfB or CP8-IsdB combining antibodies against *S. aureus* skin abscess formation. Mice were passively immunized with 300 µl of antisera mixed together and administered 24 h and 4 h prior to challenge with indicated *S. aureus* strains mixed with 60–87 µm dextran beads. The CFU/abscess recovered in mice vaccinated show a significant reduction compared to the NRS group (p values determined by Mann-Whitney test).

Since our in vitro OPKA showed that in spite of use of the 9GlcNH_2_ glycoform to induce antibody to PNAG, the antibodies to the different surface CP and PNAG polysaccharides interfered with each other's functional activity, we sought to determine if this interference affected the in vivo protection apparently mediated by antibody to IsdB. Thus, we passively immunized mice with antibodies to 9GlcNH_2_-Hla, CP5-ClfB or CP8-IsdB that were mixed together. In this setting the combination of antibodies raised against the three conjugate vaccines still significantly reduced the bacterial counts in abscess of mice challenged with either S. aureus strain Newman (P = 0.0019), PS80 (P = 0.0070) or LAC (P = 0.0068) (Fig. 6), indicating that the efficacy of antibody to the IsdB antigen was still manifest in a setting wherein the antibodies to the surface carbohydrate antigens would not be expected to be active.

### Passive immunization with anti-CP5-ClfB protects mice against *S. aureus* nasal colonization

**Figure 7:**
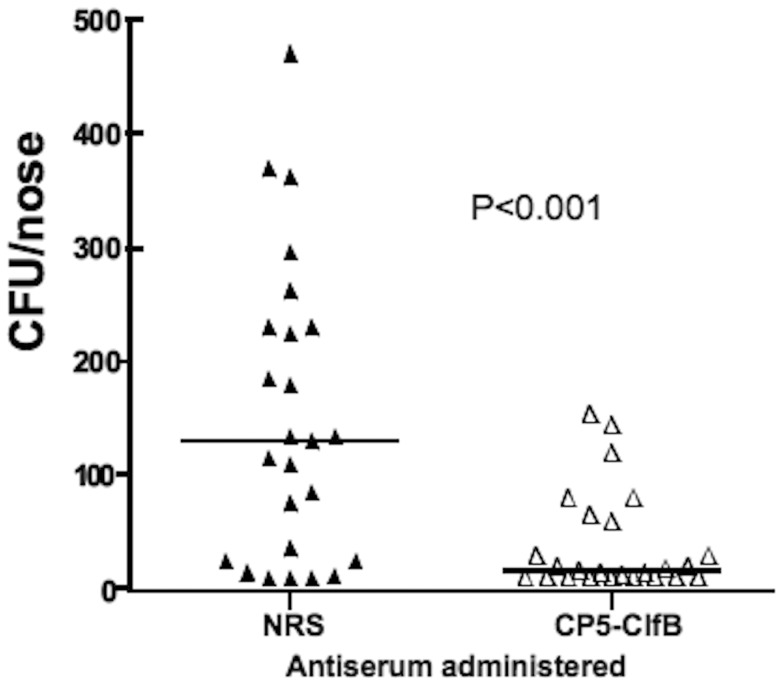
Passive immunization CP5-ClfB antibodies reduced *S. aureus* Newman nasal colonization. Mice were vaccinated IP with 300 µl of antisera 14 h prior to inoculation with 10^9^ CFU. Dots indicate the number of CFU/nose of individual mice, lines the group median, P value by Mann-Whitney U test.

Previous studies demonstrated that a S. aureus clfB mutant showed reduced nasal carriage compared to the parent strain [16] and antibody to ClfB reduced levels of bacterial colonization in the mouse nose. Because ClfB is maximally detected on the staphylococcal cells only during the logarithmic phase of growth [48], we inoculated mice with S. aureus Sm^r^ Newman grown in TSB to mid-log phase (OD_600_ nm of 0.5). Passive immunization experiments administering polyclonal anti-CP5-ClfB 14 h prior to inoculation with S. aureus Sm^r^ Newman significantly reduced the CFU/nose compared to the mice receiving NRS (P = 0.0011) (Fig. 7), indicating that conjugation of ClfB to CP5 retained its ability to induce colonization-inhibition antibodies. Attempts to determine the efficacy of antibody to ClfB in reducing nasal colonization by the other strains were thwarted by the inability of *S. aureus* PS80 or LAC to maintain nasal colonization in mice over a 5-day period.

## Discussion

Currently, the lack of requisite knowledge of effective acquired immunity in humans to S. aureus complicates the endeavor to design and evaluate an appropriate vaccine for this pathogen. As a result both laboratory investigations [4], [10], [16], [17], [18], [20], [50], [51] and human clinical trials [3], [6], [52] have evaluated vaccines based primarily on efficacy of active or passive immunization in animals and supported by some correlative in vitro functional assays such as OPK or inhibition of adherence to host tissues. To date, human studies have only been carried out by vaccination against single bacterial components and not utilized multivalent component vaccines, which have been proposed to potentially be more effective vaccines against microbial pathogens. However appealing this concept is, nonetheless there are no data to indicate if such an approach is likely to be feasible for *S. aureus* due to the aforementioned lack of knowledge of the molecular and/or cellular basis for effective human immunity to this pathogen.

Thus, at this stage, production and characterization of antigenic components for a *S. aureus* vaccine is still based on empirical approaches. Therefore, we chose to incorporate into our candidate vaccine the three known major surface polysaccharides, PNAG, CP5 and CP8, as representative of antigens that have been used successfully in other bacterial vaccines [53], [54], and three proteins, non-toxic Hla, IsdB and ClfB, due to support from other studies of protective efficacy in animal settings [5], [16], [21]. In addition, PNAG was tested because of the range of bacterial pathogens that produce this antigen, making it an attractive vaccine candidate due to its potential to protect against multiple, important human pathogens [11], [12], [55], [56], [57], [58], [59]. Of primary concern was whether the use of Hla (H35L), ClfB N region (amino acids 44 to 542) or IsdB as carrier proteins for carbohydrate antigens would result in induction of antibodies active against the native proteins expressed by *S. aureus*.

The results reported indicate that individual antisera raised in rabbits had good OPKA targeting the CP and PNAG antigens and also reduced infection in mouse models of pneumonia, skin abscesses, and in the case of antibody to CP5 and ClfB, nasal colonization. Thus, all three of the carrier proteins used showed themselves capable of promoting antibody to polysaccharide antigens when incorporated into conjugate vaccines. Unfortunately, we also found that use the 9GlcNH_2_ glycoform of PNAG to induce antibody to this antigen, as well as use of different carrier proteins, did not abrogate the interference in OPK when the antibodies raised to PNAG and CP were both present, as observed previously [15]. Given that the prior results demonstrating interference were obtained using an 85% deacetylated glycoform of native, high molecular-weight PNAG [15] in a conjugate vaccine, we had hoped that use of a smaller molecule completely devoid of acetates for immunization might overcome this interference, but this was not the case. Also, there was some expectation using different carrier proteins might have induced antibodies to PNAG and/or CP antigens with non-interfering idiotypes, as a recent study [60] showed that the specificity of MHC class II antigen presentation by carbohydrate-reactive B cells of carbohydrate-protein conjugate vaccines to responsive helper T cells was dependent upon the linkage of the polysaccharide and the carrier protein. Interference between antibody to PNAG and CP antigens was observed only when antisera were present at specific ratios because some sera have a high enough OPKA such that the activity can only inhibited when the interfering serum is diluted to achieve specific ratios with the competitor antisera. However, in considering how this might impact use of both PNAG and CP antigens in a human vaccine, the variability in individual responses likely would mean that many humans could produce antibody to PNAG and *S. aureus* CP antigens in proportions resulting in interference with the individual opsonic and protective activity. We have documented that this phenomenon occurs in serum from a high proportion of humans recovering from *S. aureus* bacteremia [15].

In terms of immunity elicited to the proteins, we found that use of the non-toxic Hla (H35L) [19] protein as a carrier for the 9GlcNH_2_ oligosaccharide of PNAG led to antibodies that neutralized the hemolytic effect of native Hla in vitro and reduced the bacterial burden of three *S. aureus* strains in a murine pneumonia model. S. aureus alpha toxin is a pore-forming cytotoxin, and previous studies indicated that an Hla (H35L) vaccine protected against lethal staphylococcal pneumonia [61], reduced the size of skin lesions and prevented dermonecrosis in mice infected subcutaneously with the epidemic USA 300 LAC community associated MRSA strain [62]. Our data indicate that Hla (H35L) is a good carrier protein for a conjugate vaccine because it retained its desirable immunogenic properties after conjugation to 9GlcNH_2_ and also there was an improved protective effect when antibody to both PNAG and Hla were administered to mice challenged with WT *S. aureus* compared to mice given this same serum and challenged with PNAG-negative *S. aureus*, wherein only the antibody to Hla would be effective in mediating protective effects.

IsdB is broadly expressed among diverse S. aureus clinical isolates and is highly conserved (94–100%) in both methicillin resistant and susceptible clinical isolates. The immunogenic role of this protein has been widely investigated. IsdB strongly induces an increase in antibody titers and is protective against abscess formation and lethal intravenous challenge [4], [63]. Here we found that conjugating CP8 to IsdB elicited antibodies that recognized native IsdB present on *S. aureus* grown in a low, but not high, iron medium and these antibodies also reduced the CFU/skin abscess caused by all three *S. aureus* strains tested, including two strains not producing CP8. When the antisera raised to the three surface polysaccharide-protein conjugates were all combined and administered to mice, the levels of bacteria in skin abscesses were still reduced, indicative of maintenance of the protective efficacy of antibody to IsdB in a setting where the antibodies to the carbohydrate antigens would be expected to lose protective efficacy due to their mutual interference. While this result might recommend IsdB as a good component of a *S. aureus* vaccine, the recent failure of this antigen as a monovalent vaccine in a clinical trial in cardiothoracic surgery patients (Merck, Intercell press release, http://www.merck.com/newsroom/news-release-archive/research-and-development/2011_0608.html, 8 June 2011), which included some suggestions of a greater, but non-significant, degree of adverse events in the IsdB-immunized individuals, likely means that further studies of this antigen in humans will be difficult to justify and undertake [64].

Clumping factor B (ClfB) has been determined to be an attractive candidate to include in a vaccine for humans as animal studies indicate antibody to this antigen reduces S. aureus nasal colonization [16]. S. aureus binds to mouse cytokeratin 10, and antibody to ClfB impairs nasal colonization, which can diminish the risk of staphylococcal infection [16]. Here we found that a CP5-ClfB conjugate vaccine induced antibodies that inhibited *S. aureus* Newman nasal colonization in mice, indicating that the conjugation of ClfB to CP5 retained epitopes capable of eliciting colonization-interfering antibody. The contribution of CP5 antibodies to the observed protection is uncertain as we were not able to obtain chronic nasal colonization in mice with a non-CP5 strain.

Overall, our findings indicate that the *S. aureus* surface polysaccharides PNAG, CP5 and CP8, when conjugated to carrier proteins Hla, IsdB or ClfB, can induce high titers of polysaccharide-specific opsonic antibody and protein specific functional antibody that individually reduced experimental S. aureus skin infection, pneumonia and nasal colonization. However, use of the synthetic oligosaccharide glycoform of PNAG and different carrier proteins did not overcome the interference in OPKA engendered when antibodies to PNAG are combined with antibody to either CP5 or CP8 [15]. This finding further indicates that a vaccine containing both *S. aureus* CP antigens and PNAG may be problematic to develop. The implications of this finding might be that if antibody to the GlcNH_2_-glycoform of PNAG is truly protective against the range of pathogens producing this antigen as a surface molecule, then vaccines raising antibody to CP antigens could interfere with the protective efficacy of antibody to PNAG. Alternately, if PNAG does not have good safety or protective efficacy in humans, then antibody to *S. aureus* CP antigens, potentially along with other vaccine components, may have high potential as a vaccine. Currently, a fully human MAb to PNAG is in phase II clinical trials (http://clinicaltrials.gov/ct2/show/NCT01389700?term=SAR279356&rank=1), and vaccines containing CP5 and CP8 conjugates along with other components are also in early clinical trials (http://clinicaltrials.gov/ct2/show/NCT01364571?term=staphylococcusaureus%26vaccine&rank=2 and http://clinicaltrials.gov/ct2/show/NCT01160172?term=staphylococcusaureus%26vaccine&rank=10), results from which may help ascertain the utility of these antigens as components of a *S. aureus* vaccine [65]. Finally, while our study focused on the role of antibodies and use of passive immunity to evaluate potential *S. aureus* vaccines, there are now some data indicating a possible role for T-cell based immunity [66], [67], particularly involving the T_H_17 pathway, in immunity to *S. aureus* infection [7]. However, it is likely most otherwise healthy humans have naturally-acquired T-cell based immunity to *S. aureus* that is contributing to resistance to infection, since individuals that lose such immunity become more susceptible to *S. aureus* infections (reviewed in [7]). Also, patients with chronic granulatomatous disease who have phagocytes unable to mediate bacterial killing due to deficiencies in the reduced NADPH-oxidase system have a high rate of *S. aureus* infections [68], indicating that classic opsonic killing, a process augmented by antibody and complement deposition onto the bacterial surface, plays a key role in human resistance to this pathogen. Thus, it is most likely that antibody mediated OPK will play an important role in vaccination against *S. aureus* infection, indicating that successful vaccine development will need to incorporate antigens capable of inducing potent OPK.

## Supporting Information

Figure S1 **Purity of indicated recombinant protein used to produce conjugate vaccines.** By analysis of the scanned gels all recombinant proteins were >95% pure.
(TIF)
