# Supplementary figures and images for: Opsonic and Protective Properties of Antibodies Raised to Conjugate Vaccines Targeting Six Staphylococcus aureus Antigens
Source: PLoS One. 2012 Oct 15;7(10):e46648. doi: 10.1371/journal.pone.0046648 (PMC3471903; doi:10.1371/journal.pone.0046648)

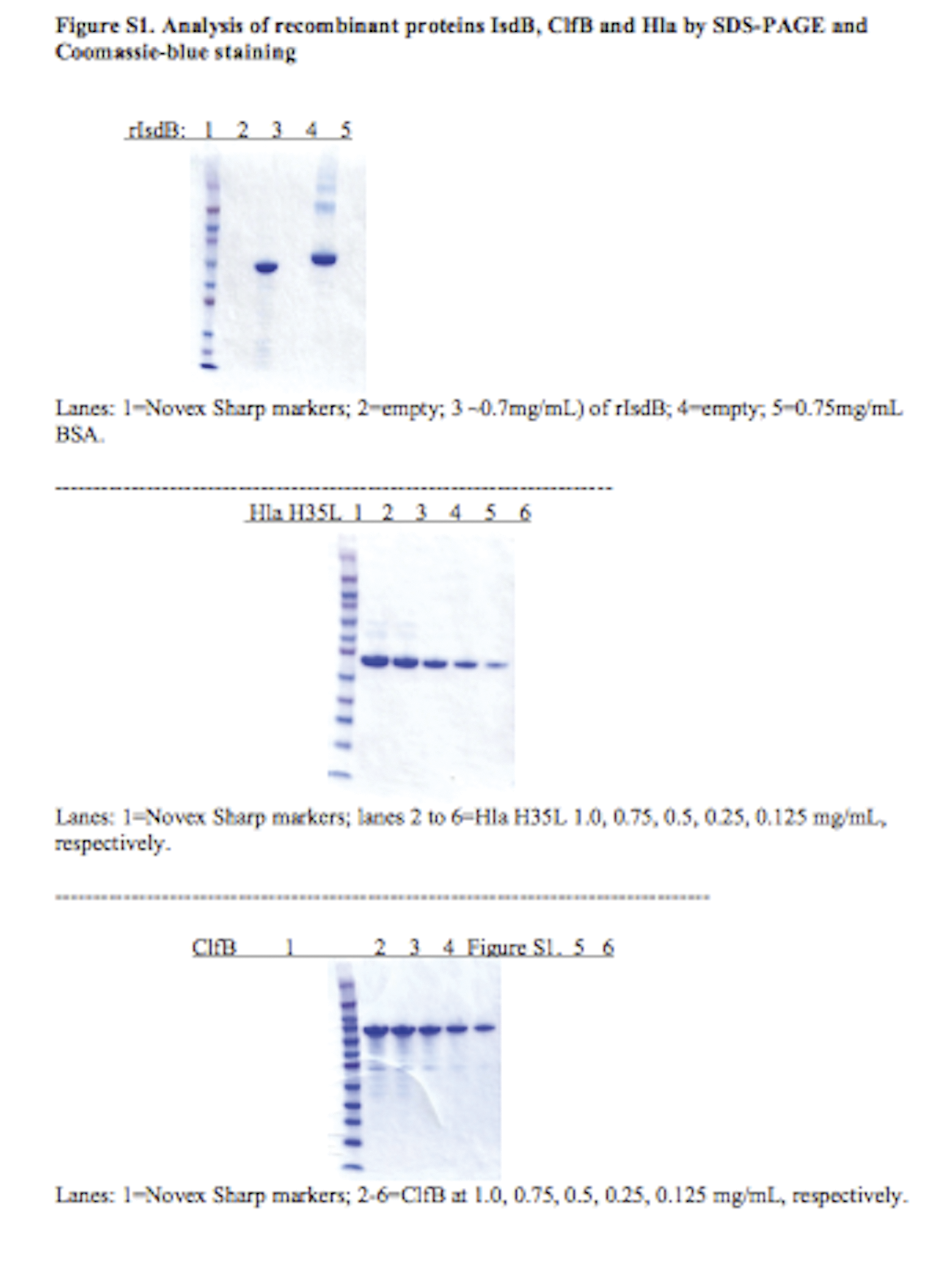

Supplement: Figure S1 — Purity of indicated recombinant protein used to produce conjugate vaccines. By analysis of the scanned gels all recombinant proteins were >95% pure. (TIF) [file pone.0046648.s001.tif]
